# Syndromic surveillance and its utilisation for mass gatherings

**DOI:** 10.1017/S0950268818001735

**Published:** 2018-06-22

**Authors:** A.C. Berry

**Affiliations:** Department of Medicine, University of South Alabama, Mobile, AL, USA

**Keywords:** Syndromic surveillance, mass gatherings, symptom checker, epidemiology, public health

## Abstract

Tremendous advancements in syndromic surveillance strategies over the last two decades, and specifically from prior mass gatherings, have been incorporated into day-to-day healthcare analysis worldwide and have left a lasting indirect impact since their inception. Mass gatherings are a daily occurrence worldwide and provide a scenario ripe for public health aims and objectives utilising syndromic surveillance. Europe is less than a decade away from hosting a colossal worldwide gathering (2024 Summer Olympics) in likely a time when the global agreement is in flux. A call to arms is needed for additional surveillance strategies incorporating mobile application symptom checker data, telemedicine, social media and social data sensing. There remains a need for an optimal combination of real-time data sensing that captures the whole population, but to reach that goal we must incorporate new advancements into baseline epidemiologic data monitoring, otherwise we will be tracking real-time mass gathering events on top of inaccurate baseline epidemiologic data.

Large mass gatherings pose a significant strain on the planning and resources of a regional event host. An influx of non-native visitors and efflux of native population combine to disrupt host baseline healthcare-related factors, including healthcare provider allocation, non-endemic seasonal and global disease variation, communication hurdles in response to a health crisis, and the strain on already limited physical resources. Preemptive event planning remains pivotal, but the real-time dynamic monitoring and response to new healthcare situations remain an imposing challenge.

Syndromic surveillance is the real-time (or near real-time) collection, analysis, interpretation and dissemination of health-related data enabling early identification of the impact (or absence of impact) of potential human public health threats that require effective public health action [1]. It relies on clinical signs, epidemiologic trends and proxy measures (e.g., absenteeism, drug sales, doctor visits) that create a provisional diagnosis (or ‘syndrome’) [1]. It has evolved over the last two decades, with each use a test and opportunity to improve its efficacy. Syndromic surveillance itself is not a ‘technology’, but instead a design of real-time disease surveillance aided by technological advances and dynamic computer algorithms.

Syndromic surveillance has been utilised in many mass gatherings to date. It has been utilised during major worldwide natural disasters (flooding, fires, volcanic eruptions) and pandemic outbreaks (2009 influenza) [2]. Syndromic surveillance has also been successfully operated during worldwide sporting events, such as the 2002 Winter Olympic Games in Salt Lake City, 2012 Summer Olympic and Paralympic Games in London, and at the 2015 Los Angeles Special Olympic World Games [1–4]. Before mainstream syndromic surveillance, ‘drop-in surveillance’ was utilised at non-sporting mass gatherings, such as large political events, national conventions, and post-sentinel event monitoring [5]. The momentum for syndromic surveillance has waxed and waned, with specific acute events and subsequent successes and failures spurring future utilisation and improvement. The 11th September 2001 attacks on the United States World Trade Center (WTC) are an example of such an event. Here, the New York City Department of Health and Mental Hygiene, combined with the United States Centers for Disease Control and Prevention (CDC) implemented a syndromic surveillance system in local emergency rooms to (1) help identify a potential secondary large-scale bioterrorist event and (2) identify local health conditions related to the initial attacks [6]. One week after the WTC attacks, a nationwide scare of anthrax outbreak via the postal system further heightened the need for syndromic surveillance refinement [6]. Syndromic surveillance is not specifically designed for terrorism or bioterrorism suspicions, but tragic events like these do exponentially escalate public demand for syndromic surveillance platforms and have contributed to its dynamic and worldwide utilisation.

Nations across Europe have built on each other's syndromic surveillance platforms. For example, the French Institute for Public Health Surveillance coordinated the Triple-S (Syndromic Surveillance Survey; 2011) to recognise potential data sources for syndromic surveillance across Europe [1]. The goal of initial syndromic surveillance measures was to incorporate epidemiologic data algorithms and daily to weekly measures of acute health-related events, to monitor deviations and daily ‘alarms’ in various categories as compared to the local epidemiologic baseline. The United Kingdom National Health Service (NHS) designed their own syndromic surveillance system, which included calls to the NHS Direct telephone health advice line in combination with a partial snapshot of UK general practitioner (GP) surveillance network monitoring the weekly number of GP consultations for various disease entities over the expected respective epidemiologic baseline [3].

The 2012 London Olympic Games offered a prime opportunity to build on prior surveillance methods and incorporate new syndromic surveillance modalities such as GP out of hours and unscheduled care visits and daily emergency department (ED) symptom and diagnostic uploads [3]. This quartet of data surveillance was able to reassure the 2012 Olympic population of encountered ‘alarm’ trends related to heat-related illness and other symptoms routinely encountered during Summer (e.g. related mainly to gastro-enteritis, possible food poisoning) [3]. Analysis did demonstrate a rise in ED visits for ‘chemicals, poisons, and overdoses, including alcohol’ and ‘acute alcohol intoxication’, all coinciding with the timing of the Olympic opening ceremony [4]. Thus, surveillance modalities not only have used in disease or symptom outbreaks but can offer event coordinators a way to monitor local-regional baseline data trends to best allocate healthcare resources ahead of time. Utilisation for mass gatherings has helped refine syndromic surveillance modalities for ongoing, day-to-day healthcare needs, such as timing and severity of seasonal illnesses, like influenza. It also sparked the need to refine baseline epidemiologic data and the importance of accurate baseline data collection strategies, with the incorporation of large academic centres and research experts, to appropriately compare event-specific data outliers and which ‘alarms’ to allocate limited resources for investigation. Since 2012, peer-reviewed scientific contributions have surfaced with a large multidisciplinary focus for further improvement [4].

With the many major mass gatherings looming and the 2024 Summer Olympic Games returning back to Europe (Paris), we must strive to align syndromic surveillance methodology with the evolving societal times and the on-demand technology-driven consumer. In 2013, the NHS performed a pilot analysis of online symptom checker data as an adjuvant to syndromic surveillance, given that roughly 90% of American and 75% of International Web users search for health information online [7]. Strong correlation between the online symptom checker and traditional telephone triage data for a number of syndromic indicators existed [7]. In addition, for some disease systems (ex. respiratory), online symptom checker data appeared to provide additional early warning over telephone triage health data [7]. The analysis also did show the use of online symptom checkers vastly outnumbered traditional telephone lines. Online symptom checkers do offer the ability to be much more cost-effective and real-time than traditional data monitoring systems. They also can be utilised globally and simultaneously and offer all users at a mass gathering a familiar healthcare data interface, regardless of local healthcare infrastructure unfamiliarity. Some can also provide healthcare advice or be uploaded simultaneously at healthcare centres. Utilising online symptom checker applications for syndromic surveillance does come with some potential ramifications, including user ability to self-control diagnosis pathways and the ability of an asymptomatic individual to navigate the application, leading to inaccurate syndromic surveillance data monitoring.

Are online symptom checkers a valid and plausible addition to mass gathering strategies? A large audit study was performed in 2015 by Semigren *et al.* analysing the potential diagnostic and triage capabilities of a multitude of online symptom checkers, with results varying vastly by diagnosis type, triage level and online symptom checker [8]. Further analysis compared symptom checkers to doctor diagnosis accuracy, with doctors outperforming symptom checkers [9]. Critiques of the original studies have called for consecutive, prospective, real-patient cases with well-validated diagnosis as a criterion paramount for the head-to-head performance of symptom checkers and physicians. In regards to both triage accuracy and diagnostic capabilities, subsequent studies have utilised real-life ED patient data and prospective, in-office, head-to-head, doctor to symptom checker comparisons, all addressing prior limitations and finding the inferior accuracy of online symptom checkers as compared with in-person doctor visits [10]. In addition, application user baseline health literacy remains a potential variable affecting navigational strategies of online symptom checker use and subsequent erratic and inconsistent data output.

The evolving consumer wants access to supplemental information and decisional software at their fingertips, and incorporation of this demand into the syndromic surveillance algorithm for future mass gatherings must be made. The times of manual data entry and tracking consumer calls as a means for syndromic surveillance will continue to fade away. The future of syndromic surveillance for mass gatherings must incorporate the on-demand consumer, utilising such surveillance modalities such as telemedicine appointments or simply mobile search engine entries. Social media – be it *Facebook, Snapchat*, *Instagram*, *Twitter*, etc – may become a pivotal data source for real-time syndromic surveillance, and its reliance on clinical signs, epidemiologic trends, and proxy measures that create a provisional diagnosis (or ‘syndrome’). People are turning to less formal means to express the aforementioned measures, personal feelings, physical symptoms and geographic location. The current combination of hashtags and location pinning on social media allow easy means to track the current geographic impact of any subject. Online symptom checkers provide an organised modality to aid in syndromic surveillance but the future will reside in more non-healthcare specific social platforms to better capture, analyse, interpret and disseminate health-related data to enable prompt identification of potential human public health impacts. Questions do remain about missing surveillance data from those populations without access to electronic means or those technologically inexperienced.

New potential syndromic surveillance sources come with their own potential injustices, unlike prior systems, which are battle-tested and have been integrated into the syndromic surveillance foundation principles of legacy building and systemic sharing of information. Tam *et al.* have proposed a research agenda for mass gatherings that encompasses a multidisciplinary evidence-based approach [11]. Stressed is the importance of strengthened public health systems and rapid responses to health risks being integrated with other important components of the overall event management. Newer surveillance systems provide an opportunity for rapid access to information, but as Tam *et al.* suggest, ‘such information is often incomplete, evolving and derived from an increasingly complex array of sources such as basic science researchers, epidemiologists, social and political scientists, and economists [11].’ Robust and accurate conglomerate mathematical modelling is needed and has the potential to directly impact public health policy and decision-making. Just as the timely on-scene allocation of first response personal or specific resources (vaccines, nourishment supplies, etc.) depends on a previously evaluated analysis and a realistic implementation trial, newer modalities such as mathematical modelling must have stringent and robust evaluation preceding their respective practicality and feasibility incorporations. These modalities must provide added value over prior systems and have the capability to assess their effectiveness for dynamic variable adjustments.

Naïve syndromic surveillance systems have vast potential to adapt to the societal demands of the individual user-controlled, on-demand and informal surveillance modalities. Utilisation of real-time syndromic surveillance data from *Facebook*, *Snapchat*, *Instagram*, *Twitter*, etc. carry the ramifications of public *vs.* private data, and what constitutes proper sharing of one's personal data. As many of these new data sensing systems are for-profit based, legalities of governmental and epidemiologic data sensing will arise and create rich controversy. Regional and cultural variations may limit widespread use of such data sensing and hasten the fluid utilisation of such powerful epidemiologic data from emergent social media systems. The aforementioned intricacies may cloud the data for those monitoring the public health aspects of mass gatherings and alter epidemiologic baselines. In the end, the optimal incorporation of newer syndromic surveillance sources will rely on a stringent evidence-based research agenda, with continuous and preemptive multi-departmental testing and reliance on a systematic approach to determine the added value of such potential innovative modalities.
